# A Benchmark for Endoluminal Scene Segmentation of Colonoscopy Images

**DOI:** 10.1155/2017/4037190

**Published:** 2017-07-26

**Authors:** David Vázquez, Jorge Bernal, F. Javier Sánchez, Gloria Fernández-Esparrach, Antonio M. López, Adriana Romero, Michal Drozdzal, Aaron Courville

**Affiliations:** ^1^Computer Vision Center, Computer Science Department, Universitat Autonoma de Barcelona, Barcelona, Spain; ^2^Montreal Institute for Learning Algorithms, Université de Montréal, Montreal, QC, Canada; ^3^Endoscopy Unit, Gastroenterology Service, CIBERHED, IDIBAPS, Hospital Clinic, Universidad de Barcelona, Barcelona, Spain; ^4^École Polytechnique de Montréal, Montréal, QC, Canada; ^5^Imagia Inc., Montréal, QC, Canada

## Abstract

Colorectal cancer (CRC) is the third cause of cancer death worldwide. Currently, the standard approach to reduce CRC-related mortality is to perform regular screening in search for polyps and colonoscopy is the screening tool of choice. The main limitations of this screening procedure are polyp miss rate and the inability to perform visual assessment of polyp malignancy. These drawbacks can be reduced by designing decision support systems (DSS) aiming to help clinicians in the different stages of the procedure by providing endoluminal scene segmentation. Thus, in this paper, we introduce an extended benchmark of colonoscopy image segmentation, with the hope of establishing a new strong benchmark for colonoscopy image analysis research. The proposed dataset consists of 4 relevant classes to inspect the endoluminal scene, targeting different clinical needs. Together with the dataset and taking advantage of advances in semantic segmentation literature, we provide new baselines by training standard fully convolutional networks (FCNs). We perform a comparative study to show that FCNs significantly outperform, without any further postprocessing, prior results in endoluminal scene segmentation, especially with respect to polyp segmentation and localization.

## 1. Introduction

**Figure 1:**
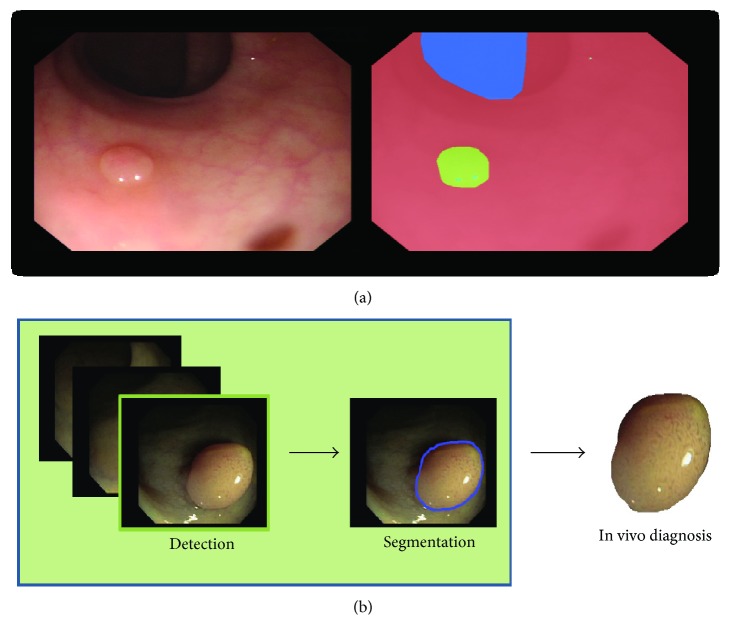
(a) Colonoscopy image and corresponding labeling: blue for lumen, red for background (mucosa wall), and green for polyp. (b) Proposed pipeline of a decision support system for colonoscopy.

Colorectal cancer (CRC) is the third cause of cancer death worldwide [1]. CRC arises from adenomatous polyps (adenomas) which are initially benign; however, over time, some of them can become malignant. Currently, the standard approach to reduce CRC-related mortality is to perform regular screening in search for polyps and colonoscopy is the screening tool of choice. During the examination, clinicians visually inspect the intestinal wall (see Figure 1(a) for an example of intestinal scene) in search of polyps. Once detected, they are resected and sent for histological analysis to determine their degree of malignancy and define the corresponding treatment the patient should undertake.

The main limitations of colonoscopy are its associated polyp miss rate (small/flat polyps or the ones hidden behind intestine folds can be missed [2]) and the fact that polyp's malignancy degree is only known after histological analysis. These drawbacks can be reduced by developing new colonoscopy modalities to improve visualization (e.g., high-definition imaging, narrow-band imaging (NBI) [3], and magnification endoscopes [4]) and/or by developing decision support systems (DSS) aiming to help clinicians in the different stages of the procedure. A clinically useful DSS should be able to detect, segment, and assess the malignancy degree (e.g., by optical biopsy [5]) of polyps during the colonoscopy procedure, following a similar pipeline to the one shown in Figure 1(b).

The development of DSS for colonoscopy has been an active research topic during the last decades. The majority of available works on optical colonoscopy are focused on polyp detection (e.g., see [6–11]), and only few works address the problems of endoluminal scene segmentation.

Endoluminal scene segmentation is of crucial relevance for clinical applications [6, 12–14]. Polyp segmentation is important to define the area covered by a potential lesion that should be carefully inspected and possibly removed by clinicians. Moreover, having a system for accurate in vivo prediction of polyp histology might significantly improve clinical workflow. Lumen segmentation is relevant to help clinicians navigate through the colon during the procedure. Additionally, it can be used to establish quality metrics related to the degree of the colon wall that has been explored, since a weak exploration can lead to polyp overlooking. Finally, specular highlights have proven to be useful in reducing polyp detection false-positive ratio in the context of handcrafted methods [15].

In recent years, convolutional neural networks (CNNs) have become a de facto standard in computer vision, achieving state-of-the-art performance in tasks such as image classification, object detection, and semantic segmentation; and making traditional methods based on handcrafted features obsolete. Two major components in this groundbreaking progress were the availability of increased computational power (GPUs) and the introduction of large labeled datasets [16, 17]. Despite the additional difficulty of having limited amounts of labeled data, CNNs have successfully been applied to a variety of medical imaging tasks, by resorting to aggressive data augmentation techniques [18, 19]. More precisely, CNNs have excelled at semantic segmentation tasks in medical imaging, such as the EM ISBI 2012 dataset [20], BRATS [21], or MS lesions [22], where the top entries are built on CNNs [18, 19, 23–25]. Surprisingly, to the best of our knowledge, CNNs have not been applied to semantic segmentation of colonoscopy data. We associate this to the lack of large publicly available annotated databases, which are needed in order to train and validate such networks.

In this paper, we aim to overcome this limitation by introducing an extended benchmark of colonoscopy images created from the combination of the two largest public datasets of colonoscopy images [6, 26] and by incorporating additional annotations to segment lumen and specular highlights, with the hope of establishing a new strong benchmark for colonoscopy image analysis research. We provide new baselines on this dataset by training standard fully convolutional networks (FCNs) for semantic segmentation [27] and significantly outperforming, without any further postprocessing, prior results in endoluminal scene segmentation.

Therefore, the contributions of this paper are twofold:

1. Extended benchmark for colonoscopy image segmentation
2. New state-of-the-art in colonoscopy image segmentation.

The rest of the paper is organized as follows. In Section 2, we present the new extended benchmark, including the introduction of datasets as well as the performance metrics. After that, in Section 3, we introduce the FCN architecture used as baseline for the new endoluminal scene segmentation benchmark. Then, in Section 4, we show qualitative and quantitative experimental results. Finally, Section 5 concludes the paper.

## 2. Endoluminal Scene Segmentation Benchmark

In this section, we describe the endoluminal scene segmentation benchmark, including evaluation metrics.

### 2.1. Dataset

Inspired by already published benchmarks for polyp detection, proposed within a challenge held in conjunction with MICCAI 2015 (http://endovis.grand-challenge.org) [28], we introduce a benchmark for endoluminal scene object segmentation.

We combine *CVC-ColonDB* and *CVC-ClinicDB* into a new dataset (*CVC-EndoSceneStill*) composed of 912 images obtained from 44 video sequences acquired from 36 patients.

- CVC-ColonDB contains 300 images with associated polyp masks obtained from 13 polyp video sequences acquired from 13 patients.
- CVC-ClinicDB contains 612 images with associated polyp and background (here, mucosa and lumen) segmentation masks obtained from 31 polyp video sequences acquired from 23 patients.

**Figure 2:**
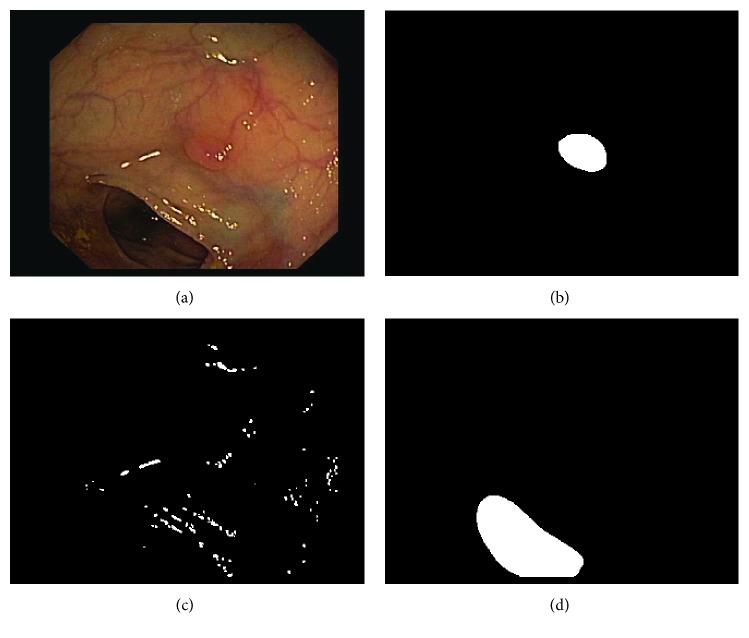
Example of a colonoscopy image and its corresponding ground truth: (a) original image, (b) polyp mask, (c) specular highlights mask, and (d) lumen mask.

We extend the old annotations to account for lumen, specular highlights with new hand-made pixel-wise annotations, and we define a void class for black borders present in each frame. In the new annotations, background only contains mucosa (intestinal wall). Please refer to Table 1 for dataset details and to Figure 2 for a dataset sample.

We split the resulting dataset into three sets: training, validation, and test containing 60%, 20%, and 20% images, respectively. We impose the constraint that one patient cannot be in different sets. As a result, the final training set contains 20 patients and 547 frames, the validation set contains 8 patients and 183 frames, and the test set contains 8 patients and 182 frames. The dataset is publicly available (http://www.cvc.uab.es/CVC-Colon/index.php/databases/cvc-endoscenestill/).

### 2.2. Metrics

We use Intersection over Union (IoU), also known as *Jaccard index*, and per pixel accuracy as segmentation metrics. These metrics are commonly used in medical image segmentation tasks [29, 30].

We compute the mean of per class IoU. Each per class IoU is computed over a validation/test set according to the following formula:

(1) $$\begin{array}{c} IoU\left(PR\left(class\right),GT\left(class\right)\right)=\frac{\left|PR\left(class\right)\cap GT\left(class\right)\right|}{\left|PR\left(class\right)\;\cup \;GT\left(class\right)\right|}, \end{array}$$

where PR represents the binary mask produced by the segmentation method, GT represents the ground truth mask, ∩ represents set intersection, and ∪ represents set union.

We compute the mean global accuracy for each set as follows:

(2) $$\begin{array}{c} Acc\left(PR,GT\right)=\frac{\#TP}{\#pixels}, \end{array}$$

where TP represents the number of true positives.

Notably, this new benchmark might as well be used for the relevant task of polyp localization. In that case, we follow Pascal VOC challenge metrics [31] and determine that a polyp is localized if it has a high overlap degree with its associated ground truth, namely,

(3) $$\begin{array}{c} IoU\left(PR\left(polyp\right),GT\left(polyp\right)\right)>0.5, \end{array}$$

where the metric is computed for each polyp independently and averaged per set to give a final score.

## 3. Baseline

CNNs are a standard architecture used for tasks, where a single prediction per input is expected (e.g., image classification). Such architectures capture hierarchical representations of the input data by stacking blocks of convolutional, nonlinearity, and pooling layers on top of each other. Convolutional layers extract local features. Nonlinearity layers allow deep networks to learn nonlinear mappings of the input data. Pooling layers reduce the spatial resolution of the representation maps by aggregating local statistics.

FCNs [19, 27] were introduced in the computer vision and medical imaging communities in the context of semantic segmentation. FCNs naturally extend CNNs to tackle per pixel prediction problems, by adding upsampling layers to recover the spatial resolution of the input at the output layer. As a consequence, FCNs can process images of arbitrary size. In order to compensate for the resolution loss induced by pooling layers, FCNs introduce skip connections between their downsampling and upsampling paths. Skip connections help the upsampling path recover fine-grained information from the downsampling layers.

We implemented FCN8 architecture from [27] and trained the network by means of stochastic gradient descent with the rmsprop adaptive learning rate [32]. The validation split is used to early stop the training; we monitor mean IoU for validation set and use patience of 50. We used a minibatch size of 10 images. The input image is normalized in the range 0-1. We randomly crop the training images to 224 × 224 pixels. As regularization, we use dropout [33] of 0.5, as mentioned in the paper [27]. We do not use any weight decay.

As described in Section 2.1, colonoscopy images have a black border that we consider as a void class. Void classes do not influence the computation of the loss nor the metrics of any set, since the pixels marked as void class are ignored. As the number of pixels per class is unbalanced, in some experiments, we apply the median frequency balancing of [34].

During training, we experiment with data augmentation techniques such as random cropping, rotations, zooming, and sharing and elastic transformations.

## 4. Experimental Results

In this section, we report semantic segmentation and polyp localization results on the new benchmark.

### 4.1. Endoluminal Scene Semantic Segmentation

In this section, we first analyze the influence of different data augmentation techniques. Second, we evaluate the effect of having different numbers of endoluminal classes on polyp segmentation results. Finally, we compare our results with previously published methods.

#### 4.1.1. Influence of Data Augmentation

Table 2 presents an analysis on the influence of different data augmentation techniques and their impact on the validation performance. We evaluate random zoom from 0.9 to 1.1, rotations from 0 to 180 degrees, shearing from 0 to 0.4, and warping with *σ* ranging from 0 to 10. Finally, we evaluate the combination of all the data augmentation techniques.

As shown in the table, polyps significantly benefit from all data augmentation methods, in particular, from warping. Note that warping applies small elastic deformation locally, accounting for many realistic variations in the polyp shape. Rotation and zoom also have a strong positive impact on the polyp segmentation performance. It goes without saying that such transformations are the least aggressive ones, since they do not alter the polyp appearance. Shearing is most likely the most aggressive transformation, since it changes the polyp appearance and might, in some cases, result in unrealistic deformations.

While for lumen it is difficult to draw any strong conclusions, it looks like zooming and warping slightly deteriorate the performance, whereas shearing and rotation slightly improve it. As for specular highlights, all the data augmentation techniques that we tested significantly boost the segmentation results. Finally, background (mucosa) shows only slight improvement when incorporating data augmentations. This is not surprising; given its predominance throughout the data, it could be even considered background.

Overall, combining all the discussed data augmentation techniques leads to better results in terms of mean IoU and mean global accuracy. More precisely, we increase the mean IoU by 4.51% and the global mean accuracy by 1.52%.

#### 4.1.2. Influence of the Number of Classes

Table 3 presents endoluminal scene semantic segmentation results for different numbers of classes. As shown in the table, using more underrepresented classes such as lumen or specular highlights makes the optimization problem more difficult. As expected and contrary to handcrafted segmentation methods, when considering polyp segmentation, deep learning-based approaches do not suffer from specular highlights, showing the robustness of the learnt features towards saturation zones in colonoscopy images.

Best results for polyp segmentation are obtained in the 2-class scenario (polyp versus background). However, segmenting lumen is a relevant clinical problem as mentioned in Section 1. Results achieved in the 3-class scenario are very encouraging, with a IoU higher than 50% for both polyp and lumen classes.

#### 4.1.3. Comparison to State-of-the-Art

Finally, we evaluate the FCN model on the test set. We compare our results to the combination of previously published handcrafted methods: [13] an energy map-based method (1) for polyp segmentation and [12] a watershed-based method (2) for lumen segmentation and [15] (3) for specular highlights segmentation.

The segmentation results on the test set are reported in Table 4 and show a clear improvement of FCN8 over previously published methods. The following improvements can be observed when comparing previously published methods to the 4-class FCN8 model trained with data augmentation: 15% in IoU for background (mucosa), 29% in IoU for polyps, 18% in IoU for lumen, 14% in mean IoU, and 14% in mean accuracy. FCN8 is still outperformed by traditional methods when it comes to specular highlight class. However, it is important to note that specular highlight class is used by handcrafted methods to reduce false-positive ratio of polyp detection, and from our analysis, it looks like the FCN model is able to segment well polyps even when ignoring this class. For example, the best mean IoU of 72.74% and mean accuracy of 94.91% are obtained by the 2-class model without additional data augmentation.

**Figure 3:**
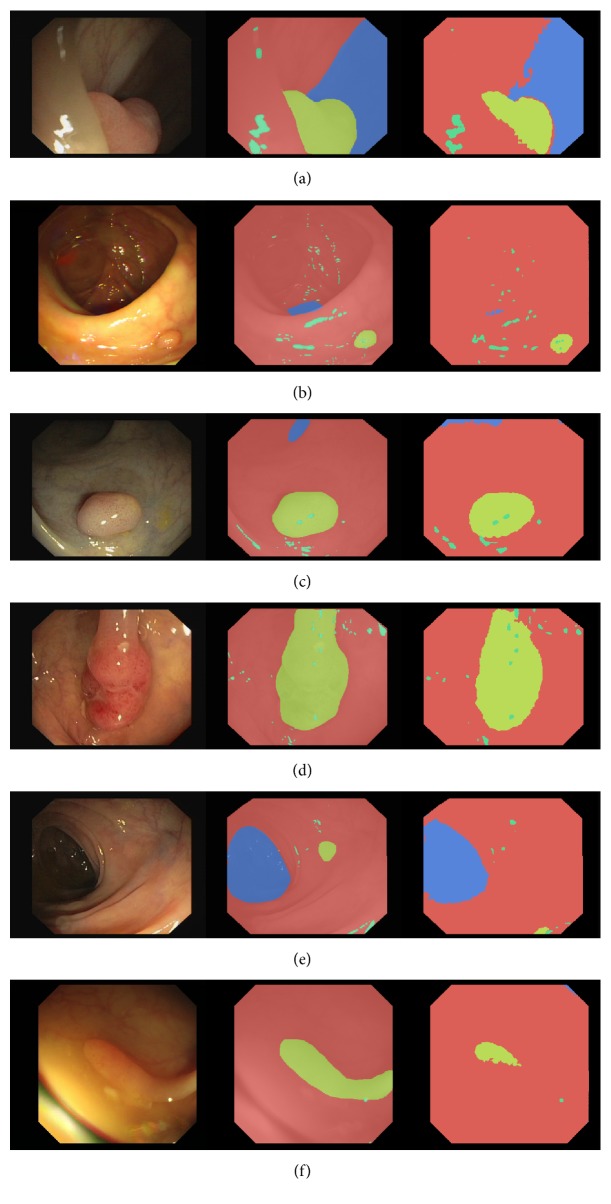
Examples of predictions for 4-class FCN8 model. Each subfigure represents a single frame, a ground truth annotation, and a prediction image. We use the following color-coding in the annotations: red for background (mucosa), blue for lumen, yellow for polyp, and green for specularity. (a), (b), (c), (d) show correct polyp segmentation, whereas (e), (d) show incorrect polyp segmentation.

Figure 3 shows qualitative results of the 4-class FCN8 model trained with data augmentation. From left to right, each row shows a colonoscopy frame, followed by the corresponding ground truth annotation and FCN8 prediction. Rows 1 to 4 show correct segmentation masks, with very clean polyp segmentation. Rows 5 and 6 show failure modes of the model, where polyps have been missed or undersegmented. In row 5, the small polyp is missed by our segmentation method while, in row 6, the polyp is undersegmented. All cases exhibit decent lumen segmentation and good background (mucosa) segmentation.

### 4.2. Polyp Localization

Endoluminal scene segmentation can be seen as a proxy to proper polyp detection in a colonoscopy video. In order to understand how well suited FCNs are to localize polyps, we perform a last experiment. In this experiment, we compute the polyp localization rate as a function of IoU between the model prediction and the ground truth. We can compute this IoU per frame, since our dataset contains a maximum of one polyp per image. This analysis describes the ability of a given method to cope with polyp appearance variability and stability on polyp localization.

**Figure 4:**
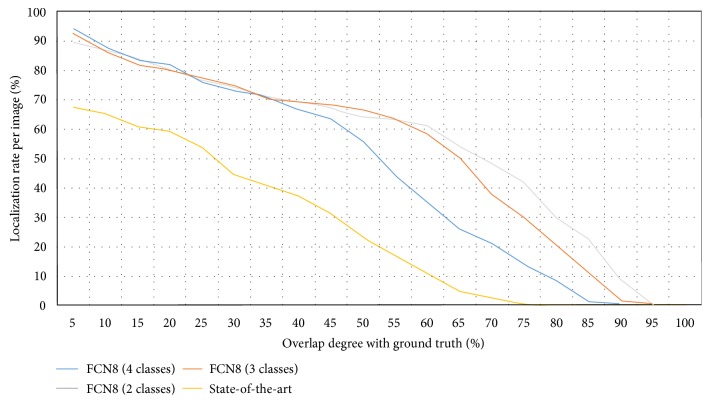
Localization rate of polyps as a function of IoU. The *x*-axis represents the degree of overlap between ground truth and model prediction. The *y*-axis represents the percentage of correctly localized polyps. Different color plots represent different models: FCN8 with 4 classes, FCN8 with 3 classes, and FCN8 with 2 classes and previously published method [13] (referred to as state-of-the-art in the plot).

The localization results are presented in Figure 4 and show a significant improvement when comparing FCN8 variants to the previously published method [13]. For example, when considering a correct polyp localization to have at least 50% IoU, we observe an increase of 40% in the polyp localization rate. As a general trend, we observe that architectures trained using a fewer number of classes achieve a higher IoU, though the polyp localization difference starts to be more visible when really high overlapping degrees are imposed. Finally, as one would expect, we observe that the architectures that show better results in polyp segmentation are the ones that show better results in polyp localization.

### 4.3. Towards Clinical Applicability

Sections 4.1.3 and 4.2 presented results of a comparative study between FCNs and previous state-of-the-art of endoluminal scene object segmentation in colonoscopy images. As mentioned in Section 1, we foresee several clinical applications, which can be built from the results of endoluminal scene segmentation. However, in order to be deployed in the exploration room, they must comply with real-time constraints apart from offering a good segmentation performance. In this case and considering videos recorded at 25 frames per second, a DSS should not take more than 40 ms to process an image in order not to delay the procedure.

Considering this, we have computed processing times for each of the approaches studied in this paper. Results are presented in Table 5.

As shown in the table, none of the presented approaches currently meet real-time constraints. Running the FCN8 inference on an NVIDIA Titan X GPU takes 88 ms per frame. Note that this could easily be addressed by taking advantage of recent research on model compression [35] by applying fancier FCN architectures that encourage feature reuse [36]. Alternatively, we could exploit the temporal component and build more sophisticated architectures that would take advantage of the similarities among consecutive frames.

Clearly, handcrafted methods take much longer to process one image. Moreover, they need to apply different methods to segment each class of interest, making them less clinically useful. Note that this is not the case for FCN-like architectures.

Despite computational constraints, FCNs' superior performance could lead to more reliable and impactful computer-assisted clinical applications, since they offer both a better performance and computational efficiency.

## 5. Conclusions

In this paper, we have introduced an extended benchmark for endoluminal scene semantic segmentation. The benchmark includes extended annotations of polyps, background (mucosa), lumen, and specular highlights. The dataset provides the standard training, validation, and test splits for machine learning practitioners and will be publicly available upon paper acceptance. Moreover, standard metrics for the comparison have been defined, with the hope to speed up the research in the endoluminal scene segmentation area.

Together with the dataset, we provided new baselines based on fully convolutional networks, which outperformed by a large margin previously published results, without any further postprocessing. We extended the proposed pipeline and used it as proxy to perform polyp detection. Due to the lack of nonpolyp frames in the dataset, we reformulated the task as polyp localization. Once again, we highlighted the superiority of deep learning-based models over traditional handcrafted approaches. As expected and contrary to handcrafted segmentation methods, when considering polyp segmentation, deep learning-based approaches do not suffer from specular highlights, showing the robustness of the learnt features towards saturation zones in colonoscopy images. Moreover, given that FCN not only excels in terms of performance but also allows for nearly real-time processing, it has a great potential to be included in future DSS for colonoscopy.

Knowing the potential of deep learning techniques, efforts in the medical imaging community should be devoted to gather larger labeled datasets as well as designing deep learning architectures that would be better suited to deal with colonoscopy data. This paper pretends to make a first step towards novel and more accurate DSS by making all code and data publicly available, paving the road for more researchers to contribute to the endoluminal scene segmentation domain.

## Figures and Tables

**Table 1:** Summary of prior database content. All frames show at least one polyp.

Database	Number of patients	Number of seq.	Number of frames	Resolution	Annotations
CVC-ColonDB	13	13	300	500 × 574	Polyp, lumen
CVC-ClinicDB	23	31	612	384 × 288	Polyp
CVC-EndoSceneStill	36	44	912	500 × 574 & 384 × 288	Polyp, lumen, background, specularity, border (void)

**Table 2:** FCN8 endoluminal scene semantic segmentation results for different data augmentation techniques. The results are reported on validation set.

Data augmentation	IoU background	IoU polyp	IoU lumen	IoU spec.	IoU mean	Acc mean
None	88.93	44.45	54.02	25.54	57.88	92.48
Zoom	89.89	52.73	51.15	37.10	57.72	90.72
Warp	90.00	54.00	49.69	**37.27**	58.97	90.93
Shear	89.60	46.61	54.27	36.86	56.83	90.49
Rotation	90.52	52.83	**56.39**	35.81	58.89	91.38
Combination	**92.62**	**54.82**	55.08	35.75	**59.57**	**93.02**

**Table 3:** FCN8 endoluminal scene semantic segmentation results for different numbers of classes. The results are reported on validation set. In all cases, we selected the model that provided best validation results (with or without class balancing).

Number of classes	IoU background	IoU polyp	IoU lumen	IoU spec.	IoU mean	Acc mean
4	92.07	39.37	**59.55**	**40.52**	57.88	92.48
3	92.19	50.70	56.48	—	66.46	92.82
2	**96.63**	**56.07**	—	—	**76.35**	**96.77**

**Table 4:** Results on the test set: FCN8 with respect to previously published methods.

	Data augmentation	IoU background	IoU polyp	IoU lumen	IoU spec.	IoU mean	Acc mean
*FCN8 performance*							
4 classes	None	86.36	38.51	**43.97**	32.98	50.46	87.40
3 classes	None	84.66	47.55	36.93	—	56.38	86.08
2 classes	None	**94.62**	50.85	—	—	**72.74**	**94.91**
4 classes	Combination	88.81	**51.60**	41.21	38.87	55.13	89.69
*State-of-the-art methods*							
[12, 13, 15]	—	73.93	22.13	23.82	**44.86**	41.19	75.58

**Table 5:** Summary of processing times achieved by the different methods studied in the paper. FCN results are the same for all four classes considered as segmentation of the four classes is done at the same time^∗^.

Method	Polyp	Lumen	Specular highlights	Background
FCN	88 ms^∗^	88 ms^∗^	88 ms^∗^	88 ms^∗^
State-of-the-art	10000 ms	8000 ms	5000 ms	23000 ms
